# Supplementary material for: Estuarine bivalve metabolic response mediated by environmental drivers
Source: PeerJ. 2025 Nov 17;13:e20357. doi: 10.7717/peerj.20357 (PMC12633146; doi:10.7717/peerj.20357)
Supplement: Supplemental Information 1 [file peerj-13-20357-s001.docx]

**Table S1.** Summary of the metabolites identified in *Austrovenus stutchburyi* across seven sampling sites in Waihi estuary.

| **Metabolites** | **Classification** |
| --- | --- |
| 2-Aminobutyric acid | Amino acid |
| Alanine | Amino acid |
| Asparagine | Amino acid |
| Aspartic acid | Amino acid |
| beta-Alanine | Amino acid |
| Carbamic acid | Amino acid |
| Cystathionine | Amino acid |
| Glutamic acid | Amino acid |
| Glutathione | Amino acid |
| Glycine | Amino acid |
| Histidine | Amino acid |
| Isoleucine | Amino acid |
| L-(-)-threo-3-Hydroxyaspartic acid | Amino acid |
| Leucine | Amino acid |
| Lysine | Amino acid |
| Methionine | Amino acid |
| N-(Carboxymethyl)-L-alanine | Amino acid |
| Ornithine | Amino acid |
| Phenylalanine | Amino acid |
| Proline | Amino acid |
| Pyroglutamic acid | Amino acid |
| Threonine | Amino acid |
| Tryptophan | Amino acid |
| Tyrosine | Amino acid |
| Valine | Amino acid |
| 10,13-dimethyl tetradecanoic acid | Fatty acid |
| 2-Hydroxyglutaramic acid | Fatty acid |
| Arachidonic acid | Fatty acid |
| DHA (Docosahexaenoic acid) | Fatty acid |
| DL-3-Aminoisobutyric acid | Fatty acid |
| DPA (Docosapentaenoic acid) | Fatty acid |
| gamma-Linolenic acid | Fatty acid |
| Margaric acid | Fatty acid |
| Myristic acid | Fatty acid |
| Palmitelaidic acid | Fatty acid |
| Pentadecanoic acid | Fatty acid |
| Stearic acid | Fatty acid |
| trans-Vaccenic acid | Fatty acid |
| 2-Aminoadipic acid | Intermediates |
| 2-Phosphoenolpyruvic acid | Intermediates |
| 4-Hydroxyphenylacetic acid | Monocarboxylic acids |
| Citric acid | Organic acid / TCA cycle |
| Fumaric acid | Organic acid / TCA cycle |
| Malic acid | Organic acid / TCA cycle |
| Succinic acid | Organic acid / TCA cycle |
| Nicotinic acid | Pyridinemonocarboxylic |

**Table S2.** Summary of PERMANOVA Pair-wise test comparing the abundance of each of the six main metabolites recorded across sites. Significant differences are shown in bold.

| Groups | Alanine | Glycine | Aspartic acid | Succinic acid | Proline | Glutamic acid |
| --- | --- | --- | --- | --- | --- | --- |
|  | P(perm) | P(perm) | P(perm) | P(perm) | P(perm) | P(perm) |
| 1, 2 | **0.016** | **0.008** | **0.023** | 0.067 | 1.000 | **0.023** |
| 1, 3 | 0.223 | 0.460 | 0.603 | **0.010** | **0.009** | **0.050** |
| 1, 4 | 1.000 | 0.564 | 0.231 | **0.031** | **0.008** | 0.064 |
| 1, 5 | 0.221 | **0.008** | **0.008** | **0.007** | **0.007** | **0.009** |
| 1, 7 | **0.008** | **0.008** | **0.007** | **0.008** | **0.009** | **0.007** |
| 1, 8 | 0.461 | **0.010** | 0.423 | **0.040** | 0.351 | 0.173 |
| 2, 3 | **0.056** | **0.026** | **0.033** | 0.138 | 0.990 | 0.301 |
| 2, 4 | 0.053 | **0.016** | 0.349 | 0.552 | 0.949 | 0.600 |
| 2, 5 | 0.056 | 0.790 | **0.007** | 0.344 | 0.559 | **0.008** |
| 2, 7 | **0.030** | **0.008** | **0.007** | 0.135 | 0.435 | **0.009** |
| 2, 8 | 0.458 | 0.822 | 0.102 | 0.975 | 0.967 | 0.251 |
| 3, 4 | 0.469 | 0.348 | 0.360 | 0.553 | 0.804 | 0.808 |
| 3, 5 | 0.983 | **0.030** | **0.007** | 0.645 | **0.007** | **0.007** |
| 3, 7 | **0.007** | **0.008** | **0.008** | 0.670 | 0.251 | **0.009** |
| 3, 8 | 0.678 | **0.035** | 0.652 | 0.182 | 0.135 | 0.577 |
| 4, 5 | 0.505 | **0.023** | **0.007** | 0.840 | **0.009** | **0.010** |
| 4, 7 | **0.016** | **0.006** | **0.010** | 0.437 | 0.222 | **0.009** |
| 4, 8 | 0.468 | **0.017** | 0.617 | 0.648 | 0.211 | 0.498 |
| 5, 7 | **0.009** | **0.007** | 0.009 | 0.500 | 0.007 | 0.061 |
| 5, 8 | 0.645 | 0.936 | 0.008 | 0.393 | 0.007 | 0.008 |
| 7, 8 | **0.017** | **0.011** | 0.008 | 0.186 | 0.059 | 0.006 |
